# Supplementary material for: Optimizing skin cancer screening with convolutional neural networks in smart healthcare systems
Source: PLoS One. 2025 Mar 25;20(3):e0317181. doi: 10.1371/journal.pone.0317181 (PMC11936426; doi:10.1371/journal.pone.0317181)
Supplement: Attachment — Submitted filename: Response to Reviewers.pdf [file pone.0317181.s003.pdf]

Optimizing Skin Cancer Screening with Convolutional Neural Networks in Smart Healthcare Systems (PONE-D-24-40790**)**

Dear Editor,
Plos One Journal

Please find below the detailed response to the Reviewers' comments. I have also uploaded the thoroughly revised version of the above-mentioned manuscript according to the Reviewer's comments, suggestions and recommendations.

Reviewers' comments:

Please include the following items when submitting your revised manuscript:

- A rebuttal letter that responds to each point raised by the academic editor and reviewer(s). You should upload this letter as a separate file labeled 'Response to Reviewers'.
- A marked-up copy of your manuscript that highlights changes made to the original version. You should upload this as a separate file labeled 'Revised Manuscript with Track Changes'.
- An unmarked version of your revised paper without tracked changes. You should upload this as a separate file labeled 'Manuscript'.

Reviewer #1:

Comment 1: The abstract of the proposed model needs to concise and brief in order to clearly demonstrate the whole manuscript.

Author Response: The abstract of the proposed model has been made concise and clear, effectively summarizing the entire manuscript.(highlighted in pdf file)

Comment 2: The related papers in the literature review needs to critically analyze.

Author Response: update the literature and critically analyzed (Highlighted in pdf file lines 84-101, lines 154-161)

Comment 3: The quality of the figures are poor, the authors needs to revised in 600 dpi for its clear visualizations.

Author Response: To improve the clarity and visual quality of the figures have been revised to 600 dpi. This higher resolution improves the visibility and elevates the overall presentation.(Fig 1-24)

Comment 4: How the authors handle the generalization of the proposed model.

Author Response: We have added figures to the manuscript that illustrate dropout, batch normalization, and regularization which play a key role in controlling the generalization of the proposed model. (Highlighted in pdf file lines 550-563, and fig.19_20)

Comment 5: For the reader concerns and to provide more details related to deep learning models, I suggest mentioning the recent deep predictors such as AIPs-SnTCN, StackedEnC-AOP, DeepAVP-TPPred, iAFPs-Mv-BiTCN, and Deepstacked-AVPs.

Author Response: To providing the more details related to deep learning models, we include an analysis of iAFPs-Mv-BiTCN in the literature review and cited. (Highlighted in pdf file lines 154-161)

Comment 6: It seems like that the authors pasted the images of the table instead creating the new table.

Author Response: We have provided the research paper in LaTeX format, with tables created based on experimental results instead of using images of tables.

Comment 7: The conclusion needs more improvement, the word "the proposed" used so many times.

Author Response: We have revised the conclusions to align with the requirements, enhancing clarity and eliminating the repetition regarding the word "proposed" (highlighted the change in pdf file. Lines 615-648)

Reviewer #2:

Comment 1: Abstract Section: Abstract should be robust in accordance to the brief introduction of the domain and topic which leads to the proposed methodology. Lastly, Research results should also be discussed briefly in comparison of state of the art.

Author Response: The abstract of the manuscript has been made concise, robust and clear to effectively summarize the entire work. (highlighted in pdf file). Results are discussed briefly in

comparison of state of the art. ((highlighted the change in pdf file. Lines 444-452, Lines 550-563, included fig 3. , table 3. , table 7.))

Comment 2: Language and Grammar: There are some language and grammatical errors, which should be corrected to maintain the manuscript's academic integrity.

Author Response: We have removed language and grammatical errors .

Comment 3: Technical Terminology: The use of technical jargon is at times inconsistent. Ensuring consistent use of technical terms would improve the manuscript's readability.

Author Response: We have used consistent technical terms to improve the manuscript's readability.

Comment 4: Graphics and Visual Aids: Figures and diagrams in the manuscript could be enhanced for clarity and better understanding. Furthermore, there should be proper explanation against each figure.

Author Response:

To improve the clarity and visual quality of the figures have been revised to 600 dpi. This higher resolution improves the visibility and elevates the overall presentation. (fig 2-24 updated) and proper explanation against each figure is also included.

Comment 5: Comparative Evaluations: Authors should include the section of comparative evaluations and they need to validate their finding with the literature.

Author Response: We included the section (fig 3. table 3, table 7.)) of comparative evaluations and validated their finding with the literature.

Comment 6: Formatting and Structure: There are many inconsistencies in the formatting and structure of the manuscript that could be rationalized for a more professional presentation. Especially, the headings and numbering should be streamlined.

Author Response: We have adjusted the numbering and headings to align with the standard paper format.

Comment 7: Explanation of Figures: Additional explanations or captions for all figures would enhance their relevance and clarity in the context of the text.

Author Response: We have improved caption , clarity and visual quality of the figures.

Reviewer #3:

The manuscript presents a valuable contribution to the field of medical imaging, particularly in the area of skin cancer detection using deep learning models. The introduction of the NGNDG-AF activation function and the integration of explainable AI techniques, such as Grad-CAM, provide significant advancements in both accuracy and transparency for the proposed model. The results are promising, showcasing high accuracy in skin cancer classification with the potential to positively impact clinical decision-making. However, there are several aspects that could be strengthened to further improve the robustness, clarity, and generalizability of the work.

Several key areas need more attention to strengthen the claims made in the manuscript:

Comment 1: While NGNDG-AF is introduced as a new activation function, a more detailed mathematical analysis is required to justify its advantages over existing functions like ReLU and ELU. Expanding the experimental comparisons with a broader range of activation functions could better demonstrate its superiority.

Author Response: A more detailed mathematical analysis is provided in the "Proposed Activation Function" section to justify its advantages over existing functions such as ReLU and ELU. Additionally, key explanations are highlighted. Also included a performance comparison of NGNDG-AF, ReLU, and ELU in terms of time consumption and accuracy. (included fig 3, lines 262-268)

Comment 2: The study is primarily focused on the HAM10000 dataset, which, while widely used, may not fully represent real-world variability in skin cancer. Including additional datasets, such as ISIC 2019, would enhance the generalizability of the results and provide stronger evidence of the model's robustness.

Author Response: In future work, we plan to extend our analysis by integrating larger and more diverse datasets, including ISIC 2019, to further validate the model's performance across different populations and skin cancer variations. This will allow us to better assess the model's applicability in clinical settings.

Comment 3: While the results are comprehensive, they could benefit from clearer organization. The improve visibility of confusion matrices would visually enhance the presentation of model performance.

Author Response: To enhance the clarity and visual quality of the confusion matrix figures, we have revised them to a resolution of 600 dpi. This increased resolution improves visibility and elevates the overall presentation. (fig 2-24) **,** confusion matrices (fig 15-18)

Comment 4: Although the validation accuracy is high, the paper lacks a detailed discussion of over fitting risks. More attention to techniques like dropout rates, regularization, or data augmentation would improve the reliability of the results and demonstrate the model's ability to generalize well to unseen data.

Author Response: We have included detailed discussion of over fitting risk and highlighted it**.** While our current study does not incorporate these some techniques, we have employed a robust validation strategy that includes Hold-out Validation, dropout rates.This approach has allowed us to achieve high validation accuracy and suggests that the model is capable of generalizing well to new data. (fig 19-20) and (lines 550-563)

Comment 5: The development of a smart healthcare system via an Android application is a noteworthy goal, but this aspect is only briefly mentioned. More details regarding the app’s architecture, usability, and implementation would provide a stronger case for its practical applicability in a healthcare setting.

Author Response: In this paper we focus on the theoretical aspects of CNN-based skin cancer classification. Similar to existing mobile applications for leaf disease detection, we envision a similar approach for skin cancer detection through an Android app. While this paper does not delve

into the app's development, we plan to address its architecture and implementation in future work. For reference, existing work(Crop AI, Leaf Doctor, AgroAl, Plantix) on leaf disease detection using mobile apps can be found here. (Note: https://agrio.app/An-app-that-identifies-plant-diseases-and-pests/, https://g.co/kgs/pnmTzvM/ ,)
