# Supplementary material for: Optimizing skin cancer screening with convolutional neural networks in smart healthcare systems
Source: PLoS One. 2025 Mar 25;20(3):e0317181. doi: 10.1371/journal.pone.0317181 (PMC11936426; doi:10.1371/journal.pone.0317181)
Supplement: Attachment — Submitted filename: Response to Reviewers_R2.pdf [file pone.0317181.s004.pdf]

Optimizing Skin Cancer Screening with Convolutional Neural Networks in Smart Healthcare Systems (PONE-D-24-40790**)**

Dear Editor,
Plos One Journal

Please find below the detailed response to the Reviewers' comments. I have also uploaded the thoroughly revised version of the above-mentioned manuscript according to the Reviewer's comments, suggestions and recommendations.

Reviewers' comments:

Please include the following items when submitting your revised manuscript:

- A rebuttal letter that responds to each point raised by the academic editor and reviewer(s). You should upload this letter as a separate file labeled 'Response to Reviewers'.
- A marked-up copy of your manuscript that highlights changes made to the original version. You should upload this as a separate file labeled 'Revised Manuscript with Track Changes'.
- An unmarked version of your revised paper without tracked changes. You should upload this as a separate file labeled 'Manuscript'.

Reviewer #2:

Reviewer: Figures and tables are not still properly formatted. There is severe visibility problems

Author Response: The figures have been formatted according to the PLOS ONE standards using the platform provided at https://pacev2.apexcovantage.com/, and the tables have been formatted according to the standard table format specified by PLOS ONE.

Journal Requirements:

Please review your reference list to ensure that it is complete and correct. If you have cited papers that have been retracted, please include the rationale for doing so in the manuscript text, or remove these references and replace them with relevant current references. Any changes to the reference list should be mentioned in the rebuttal letter that accompanies your revised manuscript. If you need to cite a retracted article, indicate the article's retracted status in the References list and also include a citation and full reference for the retraction notice.

Author Response: The following changes in reference list have been made for the manuscript:

- **References 1 , 16, 22, 27**
